# Functional Limitations, Types of Social Support, and Depressive Symptoms in Young-Old and Old-Old People in Korea

**DOI:** 10.1093/geroni/igaf122.1163

**Published:** 2025-12-31

**Authors:** Haa-young Cho, Jung-Hwa Ha

**Affiliations:** Seoul National University, Seoul, Republic of Korea; Seoul National University, Seoul, Republic of Korea

## Abstract

Instrumental Activities of Daily Living (IADLs) are essential for older adults to maintain independence and a good quality of life. The purpose of this study is to examine the effect of IADLs on depressive symptoms and moderating effects of different types of social support among young-old and old-old people. Data from the 8th and 9th waves of the Korean Longitudinal Study of Ageing were analyzed, categorizing individuals as the young-old, aged from 65 to 74(n = 1,808) or the old-old, aged 75 or older(n = 1,689). IADLs included 10 tasks such as grooming, household chores, and taking medications. Social support was classified into four types: emotional, informational, instrumental, and appraisal support. Depressive symptoms were assessed using 10 CES-D items. Hierarchical multiple regression analysis was conducted. First key finding is that in the young-old group, greater IADL limitations were significantly associated with higher depressive symptoms. However, informational support buffered IADL limitations’ negative impact-individuals with the same level of IADL limitations experienced fewer depressive symptoms if they had greater informational support. Next, in the old-old group, although the direct effect of IADL limitations on depressive symptoms was not statistically significant, the negative effect of IADL limitations on depressive symptoms was significantly alleviated among those with greater instrumental support. These results suggest different types of social support may protect different age groups from a same stressor. Thus, to protect mental health of older people, it is essential not only to provide social support but also to consider what type of support is appropriate for each age group.

